# Bi-modal ultrasound radiomics and habitat analysis enhanced the pre-operative prediction of axillary lymph node burden in patients with early-stage breast cancer

**DOI:** 10.3389/fonc.2025.1607442

**Published:** 2025-05-29

**Authors:** Jing Xu, Pan Qi, Xiaoyan Ou, Qiaoxin Zhong, Zhen-Wen Chen, Yin Wang, Aijiao Yi, Bin Wang

**Affiliations:** ^1^ Department of Medical Imaging, Yueyang Central Hospital, Yueyang, China; ^2^ Department of medical ultrasound, Yueyang Central Hospital, Yueyang, China; ^3^ Department of Artificial Intelligence, Julei Technology Company, Wuhan, China

**Keywords:** habitat analysis, shear wave elastography, radiomics, early-stage breast cancer, lymph node burden

## Abstract

**Objective:**

This study aimed to evaluate the value of habitat analysis based bi-modal ultrasound radiomics in predicting axillary lymph node (ALN) status in patients with early-stage breast cancer, and find a non-invasive and accurate method to predict ALN status.

**Materials and methods:**

A total of 206 patients with 206 breast lesions were enrolled in this study from July 2019 to December 2023. All patients were randomly divided into training cohort (165 patients) and test cohort (41 patients). The feature extraction was manually delineated with ITK-SNAP software, while a K-means clustering algorithm was employed for the segmentation of sub-regions, with the number of clusters ranging from 2 to 10. Radiomic features were extracted separately from the subregions of B-mode ultrasound (BMUS) and shear wave elastography (SWE) images after habitat generation. These modality-specific features were then combined. Eleven machine learning models were used to build models, including support vector machines (SVM), k-nearest neighbor (KNN), RandomForest (RF), ExtraTrees, XGBoost, light gradient boosting machine (LGB), NaiveBayes, AdaBoost, GradientBoosting, LR and MLP. Prediction performance was compared among clinicopathological model, omics models and habitat models.

**Results:**

According to the habitat analysis results of K clustering for BMUS and SWE, the omics features of 4 subregions for BMUS images and the 5 subregions for SWE images were extracted respectively. Compared the prediction performance of the clinicopathologic (C) risk factors model, habitat and omics models in the test cohort, NaiveBayes model based on SWE habitat achieved the highest prediction performance with AUC of 0.953 (95% CI: 0.893, 1.000)

**Conclusion:**

Habitat analysis based on ultrasound might be a potential method to visualize the intratumoral heterogeneity of breast lesions. The machine learning models based on SWE radiomics with habitat analysis could enhance the ability of prediction lymph node burden in patients with early-stage breast cancer, which could be a promising approach to make clinical decisions.

## 1 Introduction

Primary breast cancer is most commonly diagnosed cancer and the second leading cause of cancer-related death among women (1). The axillary lymph node (ALN) status in patients with breast cancer is critically important for treatment decisions and prognosis. Reliable evidence from the American College of Surgeons Oncology Group Z0011 (ACOSOG Z0011) randomized trial revealed that early-stage breast cancer patients with one or two metastatic sentinel lymph nodes can be spared ALN dissection (ALND) (2). Thus, accurate prediction of limited nodal burden (0–2 metastatic ALNs) and high nodal burden (≥3 metastatic ALNs) before surgery is very important.

B-mode ultrasound (BMUS) remains the first-line imaging modality for assessing axillary lymph node (ALN) status in clinical practice. However, its reliance on morphological features alone - such as cortical thickness and hilum preservation - results in suboptimal diagnostic accuracy. Previous studies have confirmed that BMUS alone provides insufficient preoperative evaluation of ALN status (3), creating a critical need for more reliable assessment methods.

Shear wave elastography (SWE) represents a significant technological advancement, combining conventional ultrasound with quantitative and qualitative tissue stiffness mapping through color-coded visualization (4). While SWE has demonstrated strong performance in differentiating benign from malignant breast lesions (5), and shows potential for predicting ALN status (3, 6), its clinical utility remains constrained by several factors. These include variability in measurement techniques, subjective region-of-interest (ROI) selection, and inconsistent interpretation of qualitative SWE patterns. These limitations highlight the need for more robust predictive approaches.

Radiomics has emerged as a powerful tool for extracting quantitative imaging features beyond human visual perception (7). While ultrasound-based radiomics has shown superior diagnostic performance compared to traditional methods, conventional radiomic approaches have a fundamental limitation - they analyze tumors as homogeneous entities, failing to capture the regional heterogeneity that characterizes breast cancer biology. This is where habitat imaging offers a paradigm shift. Unlike conventional radiomics, habitat analysis partitions tumors into biologically distinct subregions (habitats) based on voxel-level similarity in imaging characteristics (8, 9). Thus, the proposed integration of BMUS and SWE data for habitat analysis is particularly compelling. BMUS provides detailed structural information about tumor morphology, SWE quantifies tissue mechanical stiffness that correlate with tumor biology. This bi-modal habitat approach could provide unprecedented insights into tumor biology and significantly improve ALN status prediction. By moving beyond whole-tumor analysis to examine biologically relevant subregions, we may finally bridge the gap between imaging findings and actual metastatic potential.

Artificial intelligence (AI) have been used to identify and diagnose breast lesions (10, 11). Machine learning (ML) is a subfield of AI, involves using statistical, mathematical, and logical methods to enable machines to learn from data, which could handle complex radiomics features effectively (12). ML models have been used in the differentiation of benign and malignant breast lesions (13), prediction of ALN metastasis (14) and response to neoadjuvant chemotherapy (15). To the best of our knowledge, the use of habitat analysis with ML based on bi-modal ultrasound radiomics for prediction of ALN status in patients with breast cancer has not yet been reported.

Habitat analysis based bi-modal ultrasound radiomics might provide more morphological and biomechanical stiffness information, which could comprehensively evaluate breast cancer and reflect intratumoral heterogeneity. Hence, this study aimed to evaluate the value of habitat analysis based bi-modal ultrasound radiomics in predicting ALN status in patients with early-stage breast cancer, and find a non-invasive and accurate method to predict ALN status, which might help surgeons select individual treatment decision and improve prognosis.

## 2 Materials and methods

This retrospective study was approved by the ethics committee of Yueyang Central Hospital.

### 2.1 Patient

**Figure 1:**
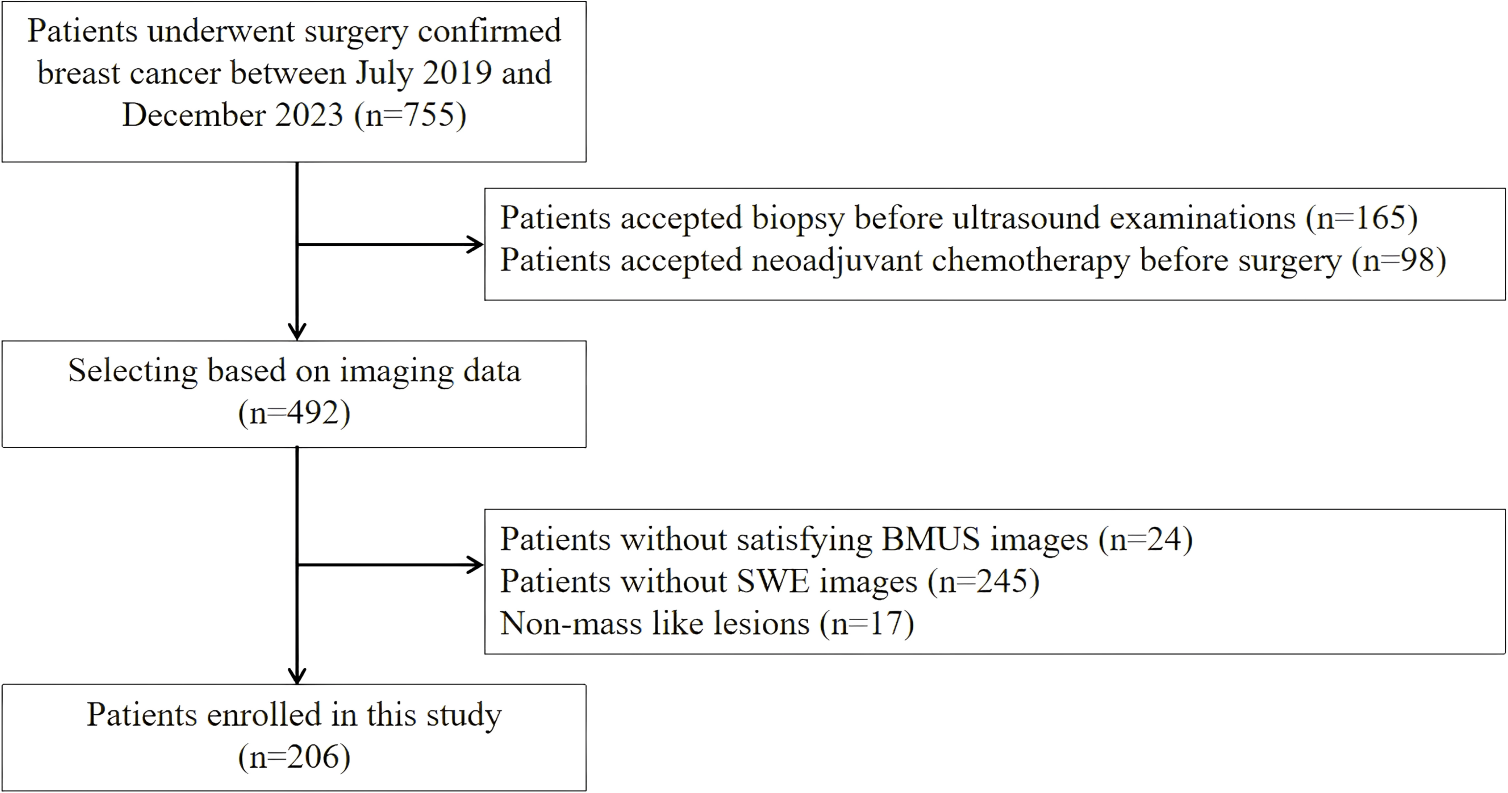
The flow chart of patient enrollment.

A total of 206 patients with 206 breast lesions were enrolled in this study from July 2019 to December 2023 (**Figure 1**). The inclusion criteria were listed as follows: (1) all breast lesions were confirmed by the final surgical pathology; (2) the breast surgery was performed within 1 month after ultrasound examination; (3) the age of all patients were over 18 years old; The exclusion criteria were listed as follow: (1) patient had accepted invasive diagnosis and therapy before ultrasound examinations; (2) the patient with unsatisfying BMUS and SWE images; (3) patients with non-mass like lesions on BMUS.

### 2.2 Ultrasound examinations

#### 2.2.1 BMUS examinations

BMUS was performed by Aixplorer ultrasound system (SuperSonic Imaging, France) with L15–4 and L10–2 linear array transducers. Patients were asked to lie in supine position with fully exposed breast and axilla. When a target breast lesion was detected, the conventional ultrasound features were recorded based on BI-RADS lexicon (2), including the shape, margin, orientation, echo pattern, posterior features and calcification. All breast masses were classified as BI-RADS 3 category (probably benign), 4a category (low suspicion for malignancy), 4b category (moderate suspicion for malignancy), 4c category (high suspicion for malignancy) or 5 category (highly suggestive of malignancy).

#### 2.2.2 SWE examinations

After conventional ultrasound, SWE was performed with L10–2 linear array transducer. Two perpendicular planes were observed for each breast mass. SWE was induced with minimal pressure, while patients were asked to hold breath for a few seconds. The region of interest should include the whole breast masses and surrounding breast tissue for at least 3mm. The stiffness was displayed with color map ranged from blue to red (0–180 KPa). The standard SWE image was obtained with several seconds of immobilization. The satisfactory SWE image was that the color fill is uniform with no mosaic artifacts. To obtain satisfactory SWE images, the SWE mode should adjust to penetration while breast masses were deeper than 1.5 centimeters.

### 2.3 Habitat generation

#### 2.3.1 Feature extraction and feature clustering

In this study, paired B-mode ultrasound and SWE images of each breast lesion were utilized for habitat analysis. The ROIs for feature extraction from the B-mode ultrasound images were manually delineated with ITK-SNAP software (3.8.0; http://www.itksnap.org), including the entire lesion area. The ROIs for the SWE image were derived from the corresponding masks of B-mode ultrasound images by shifting the XY coordinates.

For each breast lesion, nine groups of clustering features were extracted for further clustering analysis. These included Gray-Level Co-occurrence Matrix (GLCM) features (contrast, dissimilarity, homogeneity, energy, correlation, angular second moment), Shannon entropy, Local Binary Pattern (LBP) features (LBP_mean, LBP_std), and shape features (closing_mean).

A K-means clustering algorithm was employed for the segmentation of sub-regions, with the number of clusters ranging from 2 to 10. The clustering index extracted from the clustering methods included the following five metrics:

##### 2.3.1.1 Calinski-harabasz score

This metric evaluates clustering performance by assessing the compactness within clusters and the separation between clusters. A higher score indicates better clustering performance. The formula is as follows:


$$\text{CH}=\frac{\text{Tr}\left({\text{B}}_{\text{k}}\right)}{\text{Tr}\left({\text{W}}_{\text{k}}\right)}\times \frac{\left(\text{N}-\text{k}\right)}{\left(\text{k}-1\right)}$$

Where ${\text{B}}_{\text{k}}$ is the between-cluster dispersion matrix, ${\text{W}}_{\text{k}}$ is the within-cluster dispersion matrix, N is the total number of data points, k is the number of clusters.

##### 2.3.1.2 Inertia (sum of squared errors within clusters)

Inertia represents the sum of squared distances between the samples and their cluster centroids. Lower inertia values indicate that the samples are closer to their centroids, implying higher intra-cluster similarity.


$$\text{Inertia}=\sum_{\text{i}=1}^{\text{k}}\sum_{\text{x}\in {\text{C}}_{\text{i}}}\|\text{x}-{\text{μ}}_{\text{i}}{\|}^{2}$$

Where $\;{\text{C}}_{\text{i}}$ is the i -th cluster, ${\text{μ}}_{\text{i}}$ is the centroid of cluster i .

##### 2.3.1.3 Davies-bouldin index

The DBI measures the quality of clustering by comparing intra-cluster similarity with inter-cluster separation. A lower DBI indicates better clustering.


$$\text{DBI}=\frac{1}{\text{k}}{\sum_{\text{i}=1}^{\text{k}}}_{\text{j}\neq 1}^{\text{max}}\left(\frac{{\text{σ}}_{\text{i}}+{\text{σ}}_{\text{j}}}{\text{d}\left({\text{μ}}_{\text{i}},{\text{μ}}_{\text{j}}\right)}\right)$$

Where $\;{\text{σ}}_{\text{i}}$ is the average distance within cluster i, $\text{d}\left({\text{μ}}_{\text{i}},{\text{μ}}_{\text{j}}\right)$ is the distance between the centroids of clusters i and j.

##### 2.3.1.4 Separation

Separation refers to the average distance between the centroids of different clusters, measuring how distinct the clusters are. Greater separation suggests more dispersed clusters.


$$\text{Separation}=\frac{2}{\text{k}\left(\text{k}-1\right)}\sum_{\text{i}=1}^{\text{k}-1}\sum_{\text{j}=\text{i}+1}^{\text{k}}\text{d}\left({\text{μ}}_{\text{i}},{\text{μ}}_{\text{j}}\right)$$

Where $\;\text{d}\left({\text{μ}}_{\text{i}},{\text{μ}}_{\text{j}}\right)$ is the Euclidean distance between the centroids of clusters i and j.

##### 2.3.1.5 Mean intra-cluster distance

The mean intra-cluster distance is the average distance between the samples and their respective cluster centroids. A smaller value indicates tighter clusters.


$$\text{Mean Intra}\_\text{cluster Distance }=\;\frac{1}{\text{N}}\sum_{\text{i}=1}^{\text{k}}\frac{1}{\left|{\text{C}}_{\text{i}}\right|}\sum_{\text{x}\in {\text{C}}_{\text{i}}}\|\;\text{x}-{\text{μ}}_{\text{i}}\|$$

Where $\;{\text{C}}_{\text{i}}$ is the number of samples in cluster i, and ${\text{μ}}_{\text{i}}\;$ is the centroid of cluster i.

#### 2.3.2 Feature extraction and selection from habitat regions

Initially, radiomic features were extracted separately from the subregions of the two types of ultrasound images after habitat generation. These modality-specific features were then combined. A Pearson correlation coefficient threshold of 0.9 was applied to filter out highly correlated features. Subsequently, Lasso regression was employed for further feature selection, with the optimal alpha coefficient determined through tenfold cross-validation in the training cohort.

After Lasso-based selection, features with coefficients smaller than a pre-set threshold (coef = 1e-6) were excluded. Very small coefficients typically arise from noise in the data and contribute little to predictive accuracy. Retaining such features could increase the model’s sensitivity to noise and impair its generalization ability. By removing noise-driven features through thresholding, the stability and generalization performance of the model are enhanced.

#### 2.3.3 Model construction and evaluation

The features selected via Lasso were then used to construct various machine learning models. The models evaluated in this study include Support Vector Machines (SVM), k-Nearest Neighbors (KNN), Random Forest (RF), Extra Trees, XGBoost, LightGBM, Naive Bayes, AdaBoost, Gradient Boosting, Logistic Regression (LR), and Multi-Layer Perceptron (MLP). Each of these models was trained using the selected features.

The dataset was randomly split into a training cohort and a test cohort in an 80:20 ratio, with 10,000 random seeds applied. The performance of the models was evaluated using several metrics: Accuracy, area under the receiver operating characteristic (ROC) curve (AUC), 95% Confidence Interval (95% CI), Sensitivity (SEN), specificity (SPE), Positive Predictive Value (PPV), Negative Predictive Value (NPV), Precision, Recall, and F1 Score. Experiments were conducted across different feature sets, including omics and habitat. The final results were selected based on performance in the test cohort.

### 2.4 Statistical analysis

Statistical analyses were conducted with R software 3.6.1 and SPSS 23.0 software (SPSS Inc.). All numerical data are expressed as the mean ± standard deviation (SD). Categorical variables were compared using the χ2 test or Fisher’s exact test, while continuous variables were compared using the independent t-test. The training cohort was used to construct the clinicopathologic model and ML-based US radiomics or habitat models for predicting ALN burden; the test cohort was used for independent validation to evaluate the prediction performance of the models. SEN, SPE, accuracy, PPV, NPV and AUC were used to evaluate prediction performance. A two-sided *p* < 0.05 was regarded as the standard for statistical significance.

## 3 Results

### 3.1 General data of breast lesions

All patients were randomly divided into training cohort (165 patients) and test cohort (41 patients). The clinical characteristics of patients between training cohort and test cohort are shown in the **Table 1**. According to the postoperative pathological results, there were no significant differences in age, maximum size of breast lesions, histological type, tumor grade, ER status, PR status, HER2 status, Ki-67 status and molecular subtype between low and high lymph node burden groups in training cohort. Clinical stage 2 and ultrasound reported positive lymph node were associated with high lymph node burden.

**Table 1:** Clinical characteristics of patients in the training and validation cohorts.

Characteristic	Training cohort (n=164)			Test cohort (n=42)		
	Low (n=111)	High (n=53)	*p*	Low (n=30)	High (n=12)	*p*
Age,y	51.33 ± 9.49	52.62 ± 12.10	0.459	50.37 ± 10.14	50.17 ± 13.18	0.833
Maximum size (mm)	20.72 ± 7.628	23.11 ± 7.08	0.056	19.8 ± 7.98	24.58 ± 6.80	0.075
Clinical T stage (%)			0.038			0.118
cT1	59	19		18	4	
cT2	52	34		12	8	
Histological type			1.000			1.000
Ductal	105	51		27	11	
others	6	2		3	1	
Tumor grade			0.858			0.668
Low (I)/intermediate (II)	79	37		25	9	
High (III)	32	16		5	3	
ER status			0.127			0.655
Negative	31	9		5	1	
Positive	80	44		25	11	
PR status			0.593			1.000
Negative	36	15		7	3	
Positive	75	38		23	9	
HER2 status			0.135			0.719
Negative	78	31		19	9	
Positive	33	22		11	3	
Ki-67 status			0.543			0.316
≤20%	58	25		13	3	
>20%	53	28		17	9	
Molecular subtype			0.060			0.650
Luminal A	30	10		6	1	
Luminal B	51	36		19	8	
HER2 postive	11	2		2	2	
Triple negative	19	5		3	1	
US-reported LN status						
Negative	84	30	0.013	24	5	0.026
Positive	27	23		6	7	

### 3.2 Radiomics extraction and features clustering

**Figure 2:**
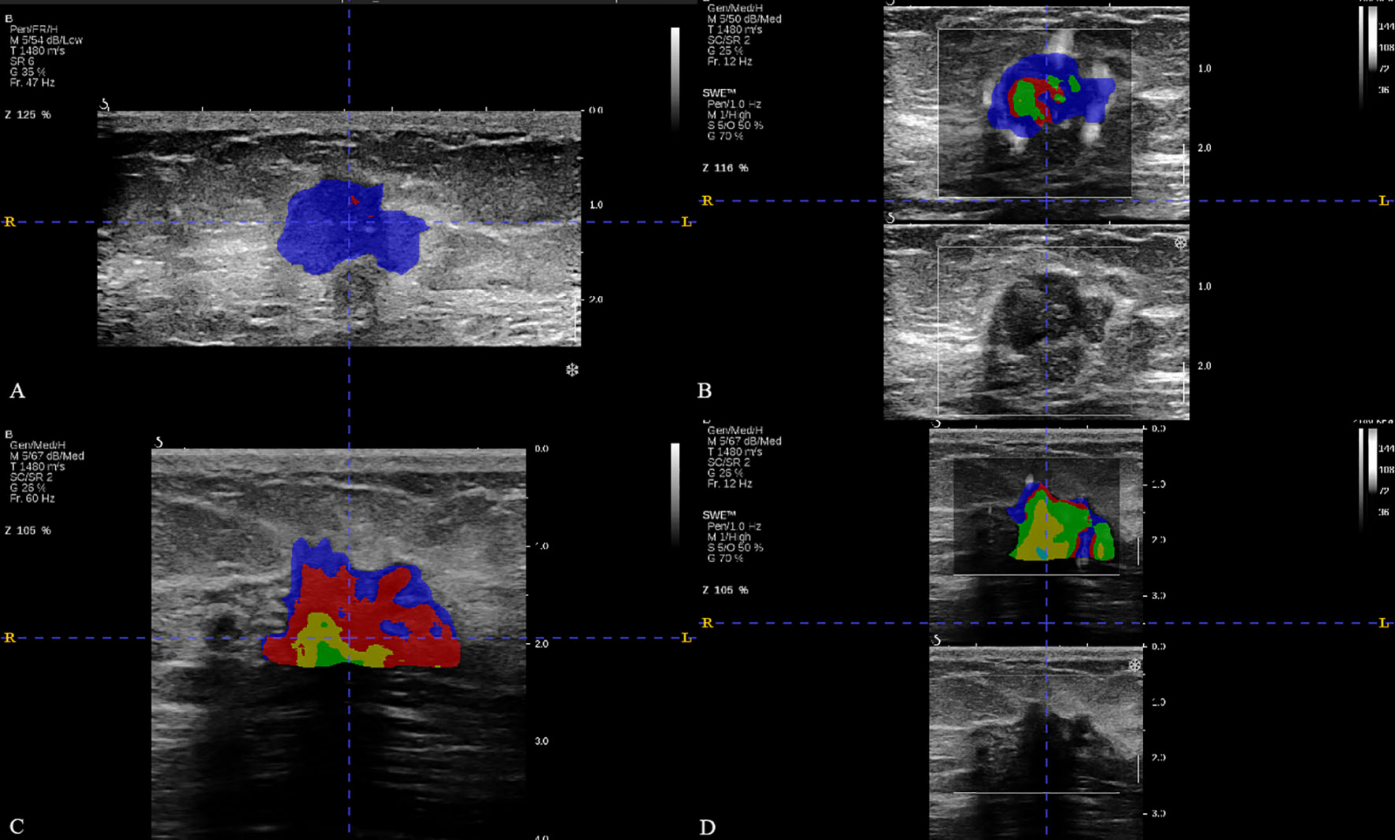
The breast cancer intratumoral heterogeneity (ITH) based on habitat in two patients with similar clinical characteristics. **(A, B)** were the habitat analyses based on BMUS or SWE for patient 1, respectively. **(C, D)** were the habitat analyses based on BMUS or SWE for patient 2, respectively. Patient 1 and patient 2 both had approximate age (48 and 50 years), the same clinical T stage, estrogen receptor (ER) status (positive), progesterone receptor (PR) status (positive), human epidermal growth factor receptor 2 (HER2) status (negative), Ki-67 index category (low proliferation) and similar BMUS or SWE features. The optimal number of clusters were 2 (BMUS) and 3 (SWE) for patient 1,while these were 4 (BMUS) and 5 (SWE) for patient 2. The final pathological surgical results were negative ALNM for patient 1 and high ALN burden for patient 2, respectively.

**Figure 3:**
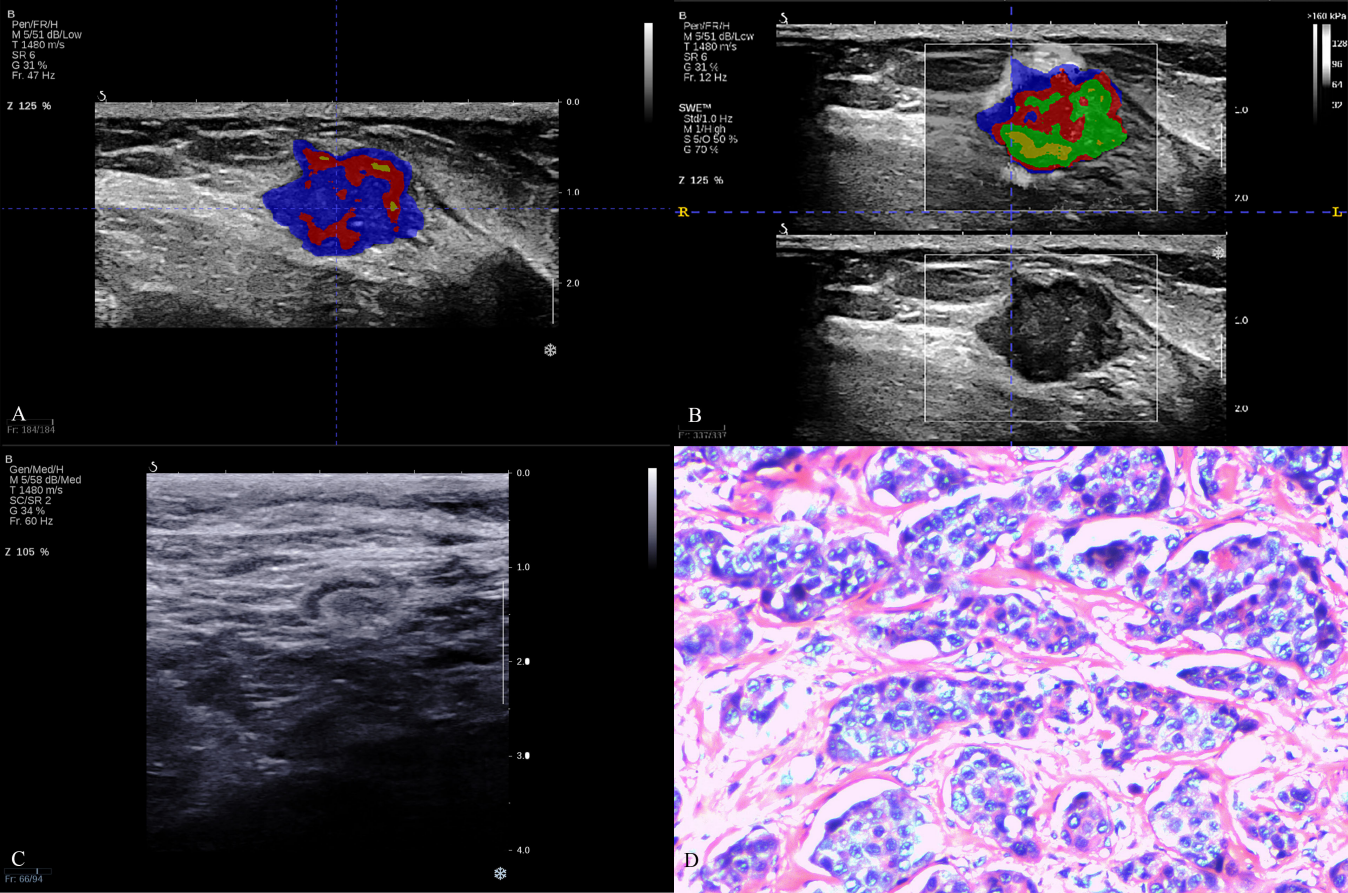
The breast cancer intratumoral heterogeneity (ITH) based on habitat, axillary lymph node on B-mode ultrasound and pathological image in a triple-negative breast cancer patient. A 62-year-old woman with breast lesion in left breast. The molecular subtype of this breast lesion was triple-negative breast cancer. The final surgical pathology indicated three lymph node metastases in the axilla, which was high lymph node burden. **(A, B)** were the habitat analyses based on BMUS or SWE, which indicated high intratumoral heterogeneity. **(C)** showed negative axillary lymph node status in B-mode ultrasound with clear cortex and medulla, normal aspect ratio and cortical thickness <3mm. **(D)** was H&E (hematoxylin and eosin) stain of a representative tumor.

A total of 927 radiomics features were extracted from each BMUS and SWE image of breast lesion, including 10 shape features, 18 first-order features, 24 GLCM features, 14 GLDM features, 16 GLRLM features, 16 GLSZM features, 5 NGTDM features, and 824 wavelet transform features. According to the quantitative analysis results of K clustering for BMUS and SWE (details are provided in Appendixes S1), the omics features of 4 subregions for BMUS images and the 5 subregions for SWE images were extracted respectively, and the features of all subregions were fused (**Figures 2**, **3**).

### 3.3 Performance of prediction models

**Figure 4:**
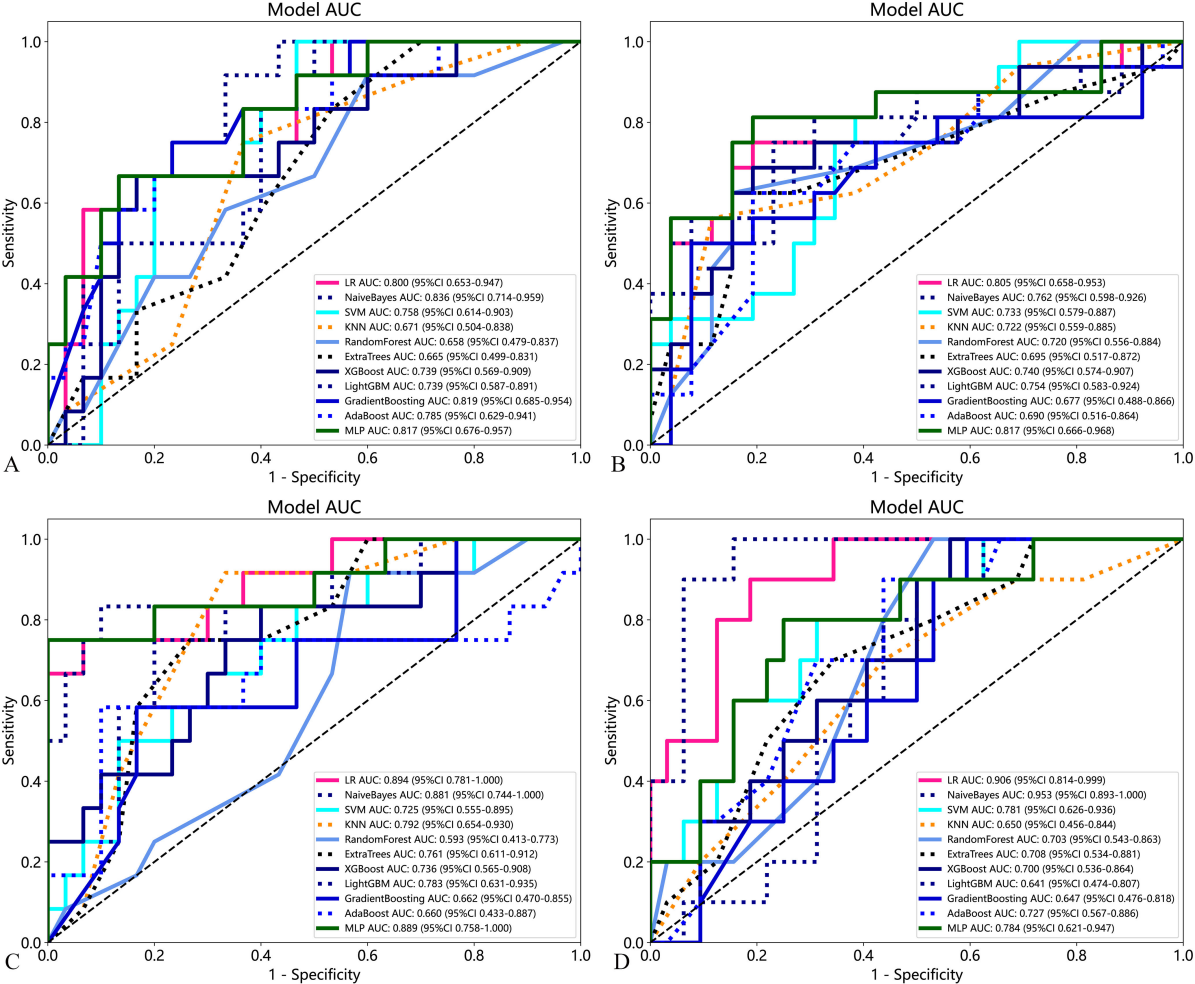
Performance of the eleven developed models based on traditional radiomics and habitat on the test cohort for predicting axillary lymph node burden in patients with early-stage breast cancer. **(A)** BMUS omics models; **(B)** SWE omics models; **(C)** BMUS habitat models; **(D)** SWE habitat models.

Eleven machine learning models based on traditional radiomics and habitat on the test cohort were established to evaluate prediction performance. In the training cohort, the prediction models were constructed with the AUC as the evaluation metrics (details are provided in Appendixes S2). The results in the test cohort were shown in **Table 2**. For BMUS based on habitat, LR achieved the highest AUC of 0.894, outperforming SVM (AUC 0.725), KNN (AUC 0.792), RF (AUC 0.593), ExtraTrees (AUC 0.761), XGBoost (AUC 0.736), LGB (AUC 0.783), NaiveBayes (AUC 0.881), AdaBoost (AUC 0.660), GradientBoosting (AUC 0.663) and MLP (AUC 0.889). For BMUS based on traditional radiomics, NaiveBayes achieved the highest AUC of 0.836, compared with other models. For SWE based on habitat, NaiveBayes achieved the highest AUC of 0.953, outperforming SVM (AUC 0.781), KNN (AUC 0.650), RF (AUC 0.703), ExtraTrees (AUC 0.708), XGBoost (AUC 0.700), LGB (AUC 0.641), LR (AUC 0.906), AdaBoost (AUC 0.727), GradientBoosting (AUC 0.647) and MLP (AUC 0.784). For SWE based on traditional radiomics, MLP achieved the highest AUC of 0.836, compared with other models (**Figure 4**).

**Table 2:** Performance of the machine learning models for the test cohort.

Model	BMUS		SWE	
	omics	habitat	omics	habitat
LR	0.800	**0.894**	0.805	0.906
NaiveBayes	**0.836**	0.881	0.762	**0.953**
SVM	0.758	0.725	0.733	0.781
KNN	0.671	0.792	0.722	0.650
RandomForest	0.658	0.593	0.720	0.703
ExtraTrees	0.665	0.761	0.695	0.708
XGBoost	0.739	0.736	0.740	0.700
LightGBM	0.739	0.783	0.754	0.641
GradientBoosting	0.819	0.663	0.677	0.647
AdaBoost	0.785	0.660	0.690	0.727
MLP	0.817	0.889	**0.817**	0.784

The bold values mean the highest AUC in all models.

### 3.4 Comparison of prediction performance for clinicopathologic risk factors model, habitat and omics models

The clinicopathologic (C) risk factors model was established based on risk factors (Clinical stage and ultrasound reported lymph node status) related to ALN burden in the univariate analysis. However, the prediction performance of C model was unsatisfactory with an SEN of 83.33%, SPE of 56.67%, PPV of 43.48%, NPV of 89.47% and AUC of 0.736 (95% CI: 0.569, 0.903). Compared the prediction performance of the clinicopathologic (C) risk factors model, habitat and omics models in the test cohort, NaiveBayes model based on SWE habitat achieved the highest prediction performance with AUC of 0.953 (95% CI: 0.893, 1.000) (**Table 3**).

**Table 3:** Comparison of prediction performance for clinicopathologic risk factors model, habitat and omics models.

Models	Method	Accuracy	AUC	95% CI	Sensitivity	Specificity	PPV	NPV
C model	risk factors	0.643	0.736	0.569 - 0.903	0.8333	0.5667	0.4348	0.8947
NaiveBayes	BMUS omics	0.714	0.836	0.714 - 0.959	0.8333	0.6667	0.5000	0.9091
LR	BMUS habitat	**0.857**	0.894	0.781 - 1.000	0.6667	0.9333	0.8000	0.8750
MLP	SWE omics	0.786	0.817	0.667 - 0.968	0.7500	0.8077	0.7059	0.8400
NaiveBayes	SWE habitat	**0.857**	**0.953**	0.893 - 1.000	0.9000	0.8437	0.6429	0.9643

The bold values mean the highest Accuracy and AUC in all models.

## 4 Discussion

In this study, eleven machine learning models for predicting lymph node burden in patients with breast cancer based on habitat and traditional radiomics extracted from BMUS or SWE. Compared using traditional radiomics, some machine learning models based on habitat could enhance the ability of prediction performance. Moreover, habitat extracted from BMUS or SWE could realize the visualization of breast tumor heterogeneity, which might be helpful explore the metastasis mechanism of breast cancer. To the best of our knowledge, this is the first study on developing machine learning models based on bi-modal ultrasound with habitat analysis for predicting lymph node burden in patients with breast cancer.

Lymph node status is an important marker for staging of breast cancer, and affecting clinical decision making. Previous study have reported that ALND can be avoided in clinical T1/T2 breast cancer patients with SLN negative as well as one or two SLN positive (2, 15). Thus, it’s important to find a non-invasive and accurate method to predict lymph node status in breast cancer patients.

Clinicalpathological risk factors have potential values in predicting ALN burden in patients with early-stage breast cancer. However, the risk factors in different studies were inconsistent. Yao et al (16) found high tumor grade and positive lymphovascular invasion (LVI) were related to sentinel lymph node metastasis (SLNM) burden. Ngai et al (17) have reported that the number of abnormal lymph nodes on axillary ultrasound was an important predictor for ALN burden. In this study, we found clinical stage and ultrasound reported lymph node status were associated with ALN burden in patients with early-stage breast cancer. However, the AUC (0.736) of C model based on risk factors for predicting ALN burden was unsatisfying, which was similar to previous study (16) with AUCs from 0.678 to 0.710. Thus, C model might be insufficient to predict ALN burden in patients with early-stage breast cancer.

Radiomics features extracted from bi-modal ultrasound images have been recognized as the core of machine learning models based on ultrasound. Radiomics features extraction and selection of previous studies mainly focused on the whole breast lesions, which neglected the intratumoral heterogeneity (18, 19). Habitat could identify subregions comprising voxels sharing similar imaging characteristics, which might share a common tumor biology, and better reflect biological behaviors. Thus, habitat analysis might be helpful to visualize the intratumoral heterogeneity of breast lesions and predict lymph node status before surgery.

In this study, we used k-means algorithm to realize feature clustering, the omics features of 4 subregions for BMUS images and the 5 subregions for SWE images were extracted respectively. We could visualize the intratumoral heterogeneity based on number of subregions in BMUS or SWE images by habitat analysis, the fewer of subregions, the lower of intratumoral heterogeneity.

Some studies have found radiomics models or machine learning models based on US radiomics analysis could be a promising predictive tool. Yu et al (20) found radiomics nomogram based on BMUS radiomics showed good performance for ALN detection (AUC 0.84 and 0.81 in the training and validation cohort). Jiang et al (21) found radiomics model based on SWE showed good discrimination for preoperative evaluation of the ALN burden in early-stage breast cancer (overall C-index 0.842 and 0.822in the training and validation set). Yao et al (16) have established four machine learning models using BMUS radiomics to predict axillary sentinel lymph node metastasis (SLNM) burden in early-stage invasive breast cancer (IBC), and found combination of clinicopathologic factors and SVM classifier model improved the predictive performance with an AUC of 0.934 in the test cohort (20).

In our study, we established eleven machine learning models based on BMUS or SWE habitat, the NaiveBayes model based on SWE habitat analysis was found to be best with AUC of 0.953, which outperformed other machine learning models for the prediction ALN burden in patients with breast cancer. Our study suggested that NaiveBayes was a robust algorithm and had strong generalization power to build habitat-based ultrasound models. Moreover, the hardness of breast cancer is not uniformly distributed, and some studies have shown that the hardness of tumor micro-environment regulates cell morphology, proliferation and metastasis (22–24). Therefore, hardness heterogeneity of tumor microenvironment quantitatively assessed by habitat-based SWE might be expected to provide reliable clues for understanding the mechanical process of breast cancer formation and development.

Several limitations associated with this study warrant mention. First, this study was a retrospective study in single center. Some unavoidable bias cannot be excluded. Second, considering the pathology heterogeneity of breast cancer, the sample size of this study was relatively small. Third, the machine learning models need to validate in multicenter external test patients. Finally, to realize SWE-based quantitative measure of breast tumor quantitative, further analysis based on habitat should be performed.

## 5 Conclusion

Habitat analysis based on ultrasound might be a potential method to visualize the intratumoral heterogeneity of breast lesions. The machine learning models based on SWE radiomics with habitat analysis could enhance the ability of prediction lymph node burden in patients with breast cancer, which could be a promising approach to make clinical decisions.

## Data Availability

The original contributions presented in the study are included in the article/**Supplementary Material**. Further inquiries can be directed to the corresponding author/s.
